# Polycystic liver disease with lethal abdominal wall rupture: a case report

**DOI:** 10.1186/s13256-021-02964-6

**Published:** 2021-08-03

**Authors:** Daichi Akuzawa, Yoichiro Uchida, Takuya Ishimura, Hiroko Kakita, Tomomi Endo, Naomi Matsuzaki, Hiroaki Terajima, Tatsuo Tsukamoto

**Affiliations:** 1 Department of Pediatrics, Tazuke Kofukai Medical Research Institute, Kitano Hospital, Osaka, Japan; 2 Department of Nephrology and Dialysis, Tazuke Kofukai Medical Research Institute, Kitano Hospital, 2-4-20 Ohgimachi, kita-ku, Osaka, 530-8480 Japan; 3 Department of Gastroenterological Surgery and Oncology, Tazuke Kofukai Medical Research Institute, Kitano Hospital, Osaka, Japan; 4 Division of Hepato-Biliary-Pancreatic Surgery and Transplantation, Department of Surgery, Graduate School of Medicine, Kyoto University, Kyoto, Japan; 5 Department of Nephrology, Graduate School of Medicine, Kyoto University, Kyoto, Japan; 6 Department of Pathology, Tazuke Kofukai Medical Research Institute, Kitano Hospital, Osaka, Japan; 7 Department of Pathology, Tenri Hospital, Nara, Japan

**Keywords:** Polycystic liver disease, Abdominal wall herniation, Case report

## Abstract

**Background:**

Polycystic liver disease is a clinical feature of autosomal dominant polycystic kidney disease, and it can sometimes cause health damage more serious than polycystic kidney. Dialysis therapy can be used for renal failure, but liver transplantation is the only method available for liver failure. Thus, giant and multiple hepatic cysts may affect mortality. However, liver transplantation is not indicated in many cases because of the preserved liver function.

**Case presentation:**

A 54-year-old Japanese woman with polycystic liver disease was transferred back to our hospital for abdominal pain caused by liver cyst infection with abdominal wall herniation. She had been diagnosed with polycystic liver disease associated with sporadic autosomal dominant polycystic kidney disease 25 years earlier. Although she had several surgical interventions to reduce her liver volume, including right hepatic lobectomy and fenestration for liver cysts in another hospital, she needed further repair of the recurrent incisional herniation with patch graft surgery using fascia lata to cover the herniation site. However, new herniation sites reemerged in the fragile abdominal wall area around the patch, and therefore, she reduced the recurrent abdominal wall herniation by herself. Recurrent intestinal obstructions were luckily released by fasting with decompression treatment via nasogastric tube insertion, but multiple skin ulcers around the enlarged hernia sac gradually developed, and ascites was extremely difficult to control with any medication. At final admission, her abdominal wall was even more prominent, causing shortness of breath, and it spontaneously ruptured many times, which was accompanied by discharge of around 5 liters of ascites each time. She died from sepsis caused by drug-resistant *Enterococcus*.

**Conclusions:**

We report a case of autosomal dominant polycystic kidney disease with ruptured abdominal wall resulting from a hepatic cyst enlargement despite multiple laparotomy operations. Throughout the entire disease course, her liver volume increased rapidly, and her quality of life was severely impaired, but she could not undergo liver transplantation after readmission to our hospital. We will discuss the therapeutic strategy for this patient, including the timing and indication for liver transplantation.

## Background

Polycystic liver disease (PLD) is characterized by multifocal cysts that grow in the liver parenchyma. Pathologically, the liver cysts are caused by intrahepatic bile duct dilation and isolation from the original ducts to form cysts. The increase in the number and growth of these cysts leads to liver enlargement, which causes compression of other abdominal organs.

PLD is genetically classified into two groups: isolated autosomal dominant polycystic liver disease (ADPLD) and PLD that is associated with autosomal dominant polycystic kidney disease (ADPKD) [1-3]. ADPKD affects 0.2% of the general population, and 75–90% of patients with ADPKD have associated PLD. However, isolated PLD affects less than 0.01% of the general population [2]. Both ADPLD and PLD are associated with ADPKD and have similar characteristics such as female dominance and the manner of inheritance. ADPLD has been linked to mutations in *PRKCSH* and *SEC63*, while the causative mutation of PLD that is associated with ADPKD is *PKD1* or *PKD2*. There is no difference in the *PKD1* and *PKD2* mutation prevalence between ethnicities, but the *PRKCH* gene mutation has been reported in Holland, Belgium, Finland, and North America, but not in Taiwan [4, 5]. The *SEC63* gene mutation has also been reported in those with a European background, but a clear localization is unknown [6]. For isolated ADPLD, the cysts are limited to the liver, with no cyst formation in other organs, whereas for PLD associated with ADPKD, patients have both liver and kidney cysts. The responsible genes of both PLD types have been identified [3, 7-10]. Growth rates of renal and hepatic cysts are not synchronized, indicating that other unknown factors that accelerate cyst growth in the liver and kidney could exist to determine the severity of PLD in ADPKD patients [11-13]. Female hormones can be an intrinsic factor that affects cyst growth because massive liver cysts at a younger age have been found mostly in women, particularly in those who experienced pregnancy [14].

As the initial treatment, partial hepatic resection is often used to control the growth of the multiple liver cysts, but limited success has been experienced, especially in cases of massive PLD. For the surgical assessment of PLD, the Gigot classification is commonly used [15]. Gigot type I patients are the best candidates for sclerotherapy and fenestration, which leads to a good prognosis [16]. However, for Gigot type III patients who are resistant to any surgery including fenestration, sclerotherapy, and partial hepatic resection, liver transplantation is the only successful treatment option [16, 17]. Treatments for Gigot type II patients remain controversial because most patients respond well to palliative surgery including sclerotherapy, fenestration, or partial hepatic resection, while some patients need a liver transplantation because of an uncontrollable liver volume increase after palliative surgery [16, 18-22]. Medical treatment including immunosuppressants is still in the development stage.

We report here a case with massive PLD in a female patient with ADPKD. For many years, multiple surgical treatments for the gradually growing liver cysts had been performed as palliative rather than radical therapy. Finally, liver growth caused uncontrollable abdominal wall ruptures before the patient died. We also discuss the timing of liver transplantation as a radical treatment along with a literature review of this disease.

## Case presentation

**Fig. 1:**
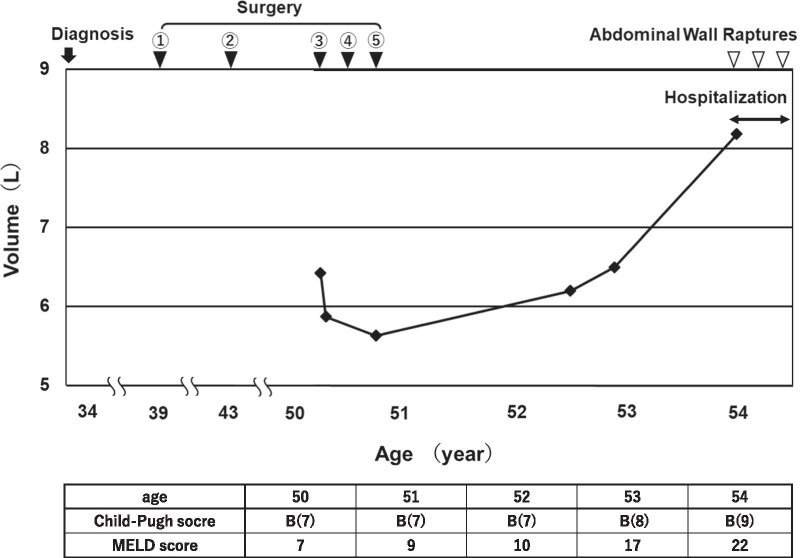
Liver volume change measured by a computed tomography (CT) image analyzer. Black triangle 1 (▼①) indicates liver fenestration of the cysts. Black triangle 2 (▼②) indicates partial right hepatic lobectomy. Surgery ① and ② were conducted at another hospital. Black triangle 3 (▼③) indicates partial liver resection, repair of the abdominal wall herniation, liver cyst fenestration, partial right hepatic lobectomy, and ovariectomy. Black triangle 4 (▼④) indicates surgery to repair the adhesive abdominal obstruction. Black triangle 5 (▼⑤) indicates surgery to repair the abdominal incisional hernia and liver cyst fenestration. White triangles (▽) indicate abdominal ruptures during the patient’s hospitalization

A 54-year-old Japanese woman presented with severe abdominal pain, and she was transferred to the emergency room at our hospital. She had given birth twice and was a housewife who had no significant problems other than high blood pressure and iron deficiency anemia. She was a never-smoker and a never-drinker and took candesartan 8 mg for hypertension. Her father had hypertension, brain infarction, and myocardial infarction. Her medical history included PLD associated with sporadic ADPKD, which was first diagnosed at 34 years of age during her second pregnancy, but she refused to undergo liver transplantation from a living donor at that time. Fenestration of liver cysts was performed at 39 years of age (Fig. 1, ▼①), and right hepatic lobectomy was performed at 44 years of age (Fig. 1, ▼②) at another hospital. Her liver size gradually increased, and she was referred to the surgical department at our hospital because of severe abdominal pain when she was 50 years old.

**Fig. 2:**
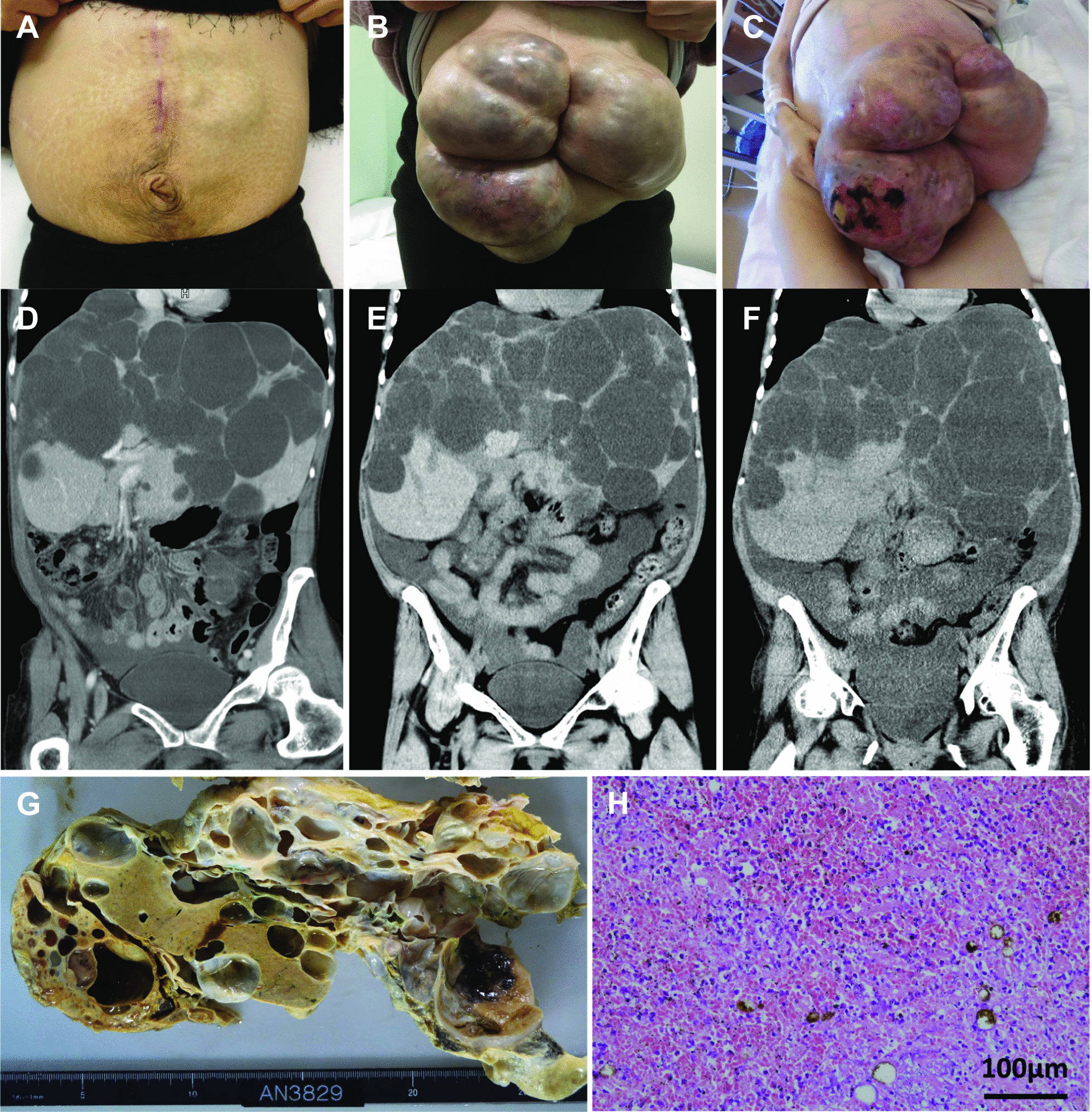
Alteration of abdominal appearances, corresponding CT images, and autopsy findings of liver parenchyma and liver cysts. Liver volume was calculated using SYNAPSE VINCENT, Fujifilm 3D image analysis system (Tokyo, Japan) using a 0.5-mm thick Axial CT image. Liver area was identified in each CT slice by hand, and integration of each slice volume led to the total volume of the cystic liver. To reduce errors, we calculated the liver volume three times and plotted the average volume. **A** and **D** Abdominal appearance and corresponding CT image after partial hepatic resection with liver cyst fenestration, repair of the abdominal wall herniation, and ovariectomy at 50 years of age. **B** and **E** Abdominal appearance and corresponding CT image at 52 years of age when the patient had an abdominal herniation. **C** and **F** Abdominal appearance and corresponding CT image on last admission at 54 years of age. Note that the prominent abdominal hernia sac can be seen with multiple skin ulcers, resulting in abdominal rupture. **G** Gross pathology of the liver parenchyma and cyst after death. The patient’s liver was filled with cysts of various sizes, leaving only a few areas of compressed parenchyma. **H** Microscopic examination of liver parenchyma. Note that many small cysts are scattered in the patient’s grossly normal liver parenchyma, and neutrophil infiltrations are found possibly because of sepsis

The cause of the abdominal pain was a strangulated hernia in the abdominal wall that included her gut and part of the liver. At that time, she underwent surgery, which was a partial hepatic resection with liver cyst fenestration, repair of the abdominal wall herniation, and ovariectomy to remove cysts that were affected by endometriosis (Fig. 1, ▼③ and Fig. 2, A and D). After surgery, she underwent abdominal incisional hernia repair twice within 6 months including partial ileectomy during the first operation (Fig. 1, ▼④) and closure of the herniation site using a native patch graft from fascia lata during the second operation (Fig. 1, ▼⑤). Despite the liver resections, her abdominal wall hernia was gradually reaggravated because of uncontrollable ascites. However, she did not require hospitalization for about 2 years because she could reduce the recurrent hernia by herself.

At the age of 52 years, she noticed severe abdominal pain unlike before, and she was transferred to our hospital. An intestinal obstruction was suspected, but her symptoms were luckily relieved by fasting with decompression treatment via nasogastric tube insertion. She asked for second opinions about liver transplantation, but she was not selected as a recipient candidate at three major liver transplant facilities on the basis of her normal liver function, Child–Pugh score (7: B), and Model for End-Stage Liver Disease (MELD) score [10] (Fig. 2, B and E). At that time, the distribution of her liver cysts had progressed to Gigot type 2.

At 54 years of age, she was readmitted to our hospital for fever, general fatigue, edema in her lower extremities, and abdominal pain. Her vital signs were as follows: body temperature 38 ℃, blood pressure 88/45 mmHg, and heat rate 109 beats/minute. Physical examination results showed conjunctival pallor, no heart murmur, no rale on lung auscultation, prominent abdominal distension with multiple skin ulcers, and edema in both lower legs. There were no abnormalities in neurological findings. Laboratory test results on admission suggested liver cyst infection (Table 1). However, the causal bacteria were not detected in her blood or in ascitic fluid culture. We treated her for a cystic infection or abscess. Tazobactam piperacillin 4.5 g was administered intravenously every 8 hours (q8h), and her fever and laboratory data [C-reactive protein (CRP) level and complete blood chemistry (CBC) test results] returned to normal within 1 month. We changed tazobactam piperacillin to amoxicillin clavulanic acid 1 g every 12 hours (q12h) because a drug allergy was suspected, but her CRP level increased again. We changed the drug treatment to meropenem 0.5 g intravenously q12h, and her CRP level started to decrease. The patient’s infection was under control in 2 weeks. Her abdominal wall was even more prominent with growing liver cysts and increasing ascites. Excessive abdominal hernia sac distention was seen around this period and led to multiple skin ulcers with oozing on the sac (Fig. 2, C and F). The severity of liver dysfunction or failure was expressed as the Child–Pugh and MELD scores, which were only B [8] and 17, respectively, until the day of her death (Fig. 1). We had no choice but to increase the amount of diuretics to control the progressive ascites that were worsening the abdominal distension. Azosemide 60 mg/day, flosemide 60 mg/day, and tolvaptan 15 mg/day were administered orally to control edema and progressive ascites that were worsening the abdominal distension. However, these medications did not relieve the patient’s symptoms. In addition, she received heparin 10,000 U/day for 20 days and apixaban 5 mg/day to prevent deep vein thrombosis for 5 months after treatment because she had difficulty moving, and her admission laboratory test results indicated that she had hypercoagulability.

**Table 1.** Laboratory data on admission

Urinalysis			Blood chemistry		
Protein	(1+)		AST	76	U/L
Sugar	(−)		ALT	64	U/L
Occult blood	(−)		ALP	1039	U/L
RBC	1–4	/HPF	γ-GTP	231	U/L
WBC	1–4	/HPF	T-Bil	1.9	mg/dL
			TP	6.7	g/dL
CBC			Alb	3.4	g/dL
RBC	386 × 10^4^	/µL	CK	81	mg/dL
Hb	9.5	g/dL	Amy	68	mg/dL
Ht	30.5	%	T-Chol	114	mg/dL
PLT	29.9	/µL	BUN	26.2	mg/dL
WBC	7900	/µL	UA	6.1	mg/dL
Neu	81.3	%	Cre	1.55	mg/dL
Lymph	8.5	%	eGFR	28	mL/min/1.73 m^2^
			Na	131	mEq/L
Coagulation			K	3.4	mEq/L
PT-INR	1.41	INR	Cl	94	mEq/L
PT (%)	48	mg/dL	Ca	9.1	mg/dL
Fibrinogen	479	mg/dL	P	3.4	mg/dL
D-dimer	5.9	μg/mL	CRP	16.83	mg/dL

*RBC* red blood cell, *Hb* hemoglobin, *Ht* hematocrit, *PLT* platelet, *WBC* white blood cell, *Neu* neutrophil, *Lymph* lymphocyte, *RT-INR* prothrombin time international normalized ratio, *TP* prothrombin time, *AST* aspartate aminotransferase, *ALT* alanine aminotransferase, *ALP* alkaline phosphatase, *γ-GTP* gamma-glutamyl transpeptidase, *T-Bil* total bilirubin *TP* total protein, *Alb* albumin, *CK* creatin kinase, *Amy* amylase, *T-Chol* total cholesterol, *BUN* blood urea nitrogen, *UA* uric acid, *Cre* creatinin, *eGFR* estimated glomerular filtration rate, *Na* sodium, *K* potassium, *Cl* chloride, *Ca* calcium, *P* phosphate, *CRP* C-reactive protein

We determined the patient’s liver volume by manually plotting the size of the liver on her CT image and reconstructing it three-dimensionally using SUNAPSE VINCENT (Fujifilm 3D Image Analysis System, Tokyo, Japan). Her liver size increased from 5.7 to 8.2 liters in 3 years, and the normal liver tissue was gradually compressed by the growing cysts (Fig. 1Ba–c). Finally, she had spontaneous abdominal wall rupture, and almost 5 liters of ascites was discharged from her abdominal cavity. After that, the ruptured abdominal wall persisted, but the ascites loss was not constant from 0.1 to 8.6 liters a day. In the last 3 months of hospitalization, a total of 2 liters or more of ascites was discharged ten times, and additional fluid supplementation was required after massive ascites loss. Antibiotics administration was also needed for comorbid peritonitis. Only a topical therapy was performed for the wound, which was a topical spray that included trafermin. The frequency of large amounts of ascites leakage had been gradually shortened, and more than 5 liters of ascites drainage was found twice a month before death. Although the abdominal distention was relieved after the rupture, her nutritional state worsened each day. She developed a high fever again after bathing. We detected *Enterococcus faecium*, *Achromobacter xylosoxidans*, *Candida tropicalis*, and *Candida glabrata* in ascitic fluid culture, while no bacteria were detected in a blood culture. Opiates were needed for unbearable abdominal wall pain. She was treated with vancomycin 0.5 g intravenously q12h, teicoplanin 400 mg intravenously every 48 hours (q48h), and linezolid 600 mg orally q12hr because the detected *E. faecium* was susceptible to only those antibiotics. However, it became difficult to continue these medications because of severe thrombocytopenia. Meropenem 0.5 g intravenously q24h and micafungin 50 mg intravenously q24h were also administered, but her infection and bleeding became uncontrollable, and she died from sepsis. Inflammatory markers such as white blood cell count and CRP level fluctuated during the patient’s disease course. However, liver enzymes indicating cholangitis showed subtle changes but no particular exacerbation in renal function. We performed an autopsy after receiving approval from her family to perform a pathological examination and their permission to publish the results. Many neutrophils had accumulated in the liver in addition to the cysts on microscopic examination (Fig. 2 G and H). The kidneys also showed typical changes in polycystic kidney disease, but no notable findings in other organs. The patient’s kidney cysts increased minimally during the disease course.

## Discussion and conclusion

There is no well-established therapy regimen for rapidly growing PLD [16, 18]. Historically, several surgical treatments have been reported for this disease, including fenestration of the liver cysts, segmental resection of the liver, and liver transplantation [22].

The Gigot classification is commonly used to determine the surgical indication of PLD [15]. In this classification, PLD is classified into three stages based on the CT image. Type I is defined as patients with a limited number (< 10) of large cysts (> 10 cm in diameter). Type II is defined as patients with diffuse involvement of the liver parenchyma by multiple medium-sized cysts with remaining large areas of noncystic liver parenchyma. Type III is defined as a severe form of PLD with massive, diffuse involvement of liver parenchyma by small- and medium-sized liver cysts and only a few areas of normal liver parenchyma between cysts [15]. This classification is convenient to classify PLD; however, three stages do not have clear border line. Therefore, it is difficult to precisely estimate prognosis and appropriate therapy only by this classification. For this reason, therapeutic strategy, especially for Gigot type II PLD, is still controversial. For type II patients, palliative surgery reduces the liver volume, but in some cases, liver cysts regrow and, ironically, these patients are no longer eligible to undergo liver transplantation because of postoperative complications including abdominal incisional hernia and adhesions. Previously, only 24% of PLD patients after fenestration and 34% of patients after hepatic segmental resection had been reported to experience liver cyst reenlargement [16]. However, if the patient has a history of multiple births, similar to our patient, worsening of the long-term prognosis should be considered. It is estimated that over 80% of patients who underwent liver transplantation were female because the cyst growth rate is relatively rapid among female patients and it has a suspected estrogen-mediated mechanism [19, 22].

To the best of our knowledge, there are no reports focusing on cyst reenlargement after palliative surgery. According to previous research, the liver volume after fenestration in PLD patients is lower than the preoperative volume for 2–8 years after fenestration regardless of the Gigot classification [15]. In our patient, the liver volume increased again to the same size within 2 years after the palliative surgery via a suspected estrogen-mediated mechanism, indicating that higher-risk patients should be identified and early liver transplantation should be recommended even if they are classified as having a lower grade using the Gigot classification. As shown in our case, because liver function is preserved until the terminal phase in PLD, PLD patients often do not satisfy the general indication for liver transplantation even in the terminal stage. Another problem is that the Child–Pugh and MELD scores are not good metrics for liver transplantation in PLD patients because they were originally designed to evaluate general liver failure (Fig. 1) [23-25].

We performed an autopsy on this patient. Her liver was filled with cysts of various sizes, leaving only few areas of compressed parenchyma (Fig. 2G). These cysts were filled with bilious viscous liquid, which indicated inflammation. Microscopic findings demonstrated that there were many neutrophils that had accumulated around the cysts, which may indicate a cyst infection, and many small cysts were scattered in her grossly normal liver parenchyma (Fig. 2H), indicating that, although her liver looked normal, it was not normal in this case. No yeast such as fungi nor purulent matter was detected in the liver or kidney. The patient’s kidney cysts increased minimally during the disease course.

Finally, we discuss the ideal timing of liver transplantation for patients who are refractory to any palliative surgeries and review the non-incisional therapeutic options. First, when is the best timing for liver transplantation for PLD patients? It seems to be hard for patients to decide in the early stage of PLD when there are fewer complications and normal liver function. Most patients choose palliative therapy at first, but some of them need liver transplantation during the disease course. We suggest performing organ transplantation before the development of an abdominal hernia because, if a mesh graft is selected instead of an autogenous patch graft, it could increase the incidence of postoperative infection, and thereby make it difficult to use immunosuppressants. Uncontrollable ascites, cyst infection, and mechanical obstruction may develop concurrently with abdominal herniation, and these complications also increase debilitation in the patient and make them ineligible for liver transplantation [26]. Combined liver and kidney transplantation might be an ideal solution for severe symptomatic PLD that is associated with ADPKD [27, 28].

We also reviewed the non-incisional therapeutics for PLD. Percutaneous transcatheter artery embolization is an option for a patient without surgical indication. In many cases of PLD that is associated with ADPKD, cyst distribution is not uniform [29]. Therefore, selective embolization is considered to be an ideal treatment. Additionally, medical therapeutics are also being developed for ADPKD and PLD. Tolvaptan, a recently approved vasopressin type 2 (V2) receptor antagonist, was approved by the United States Food and Drug Administration (USFDA) to reduce the volume of the kidney in ADPKD. However, this is not effective for suppressing liver cyst growth because the V2 receptor is not expressed in the liver cysts [3]. Sirolimus, an immunosuppressive agent for organ transplantation, might decrease the liver volume through a potential antiproliferative effect [30, 31]. Octreotide, a somatostatin analog, could reduce kidney and liver cyst fluid accumulation among patients with ADPKD or isolated PLD [31-33]. Some current drugs under development are very expensive, but they can provide options for developing drugs that can inhibit the growth of both liver and kidney cysts in PLD patients. In the present situation, early liver transplantation is the only radical treatment, but the development of these noninvasive therapeutics will provide us with new treatment strategies in the future.

We report a case of rapidly growing PLD associated with ADPKD. In a female patient, especially in multiparous patients, it may be desirable to perform an early liver transplantation instead of palliative surgery because the liver cysts may grow rapidly. The development of new drugs to reduce liver and kidney cysts is expected for these patients with PLD.

## Data Availability

Not applicable

## Abbreviations

PLD: Polycystic liver disease
ADPKD: Autosomal dominant polycystic kidney disease
V2: Vasopressin type 2
